# Multiple Disruptions of Glial-Neuronal Networks in Epileptogenesis That Follows Prolonged Febrile Seizures

**DOI:** 10.3389/fneur.2021.615802

**Published:** 2021-02-18

**Authors:** Gary P. Brennan, Megan M. Garcia-Curran, Katelin P. Patterson, Renhao Luo, Tallie Z. Baram

**Affiliations:** ^1^Departments of Anatomy/Neurobiology, Pediatrics, and Neurology, University of California, Irvine, Irvine, CA, United States; ^2^School of Biomolecular and Biomedical Science, University College Dublin, Dublin, Ireland; ^3^FutureNeuro Research Centre, Royal College of Surgeons Ireland, Dublin, Ireland

**Keywords:** epilepsy, microRNA, microglia, astrocyte, cytokines, neuroinflammation, high mobility group box 1, prostaglandins

## Abstract

**Background and Rationale:** Bi-directional neuronal-glial communication is a critical mediator of normal brain function and is disrupted in the epileptic brain. The potential role of aberrant microglia and astrocyte function during epileptogenesis is important because the mediators involved provide tangible targets for intervention and prevention of epilepsy. Glial activation is intrinsically involved in the generation of childhood febrile seizures (FS), and prolonged FS (febrile status epilepticus, FSE) antecede a proportion of adult temporal lobe epilepsy (TLE). Because TLE is often refractory to treatment and accompanied by significant memory and emotional difficulties, we probed the role of disruptions of glial-neuronal networks in the epileptogenesis that follows experimental FSE (eFSE).

**Methods:** We performed a multi-pronged examination of neuronal-glia communication and the resulting activation of molecular signaling cascades in these cell types following eFSE in immature mice and rats. Specifically, we examined pathways involving cytokines, microRNAs, high mobility group B-1 (HMGB1) and the prostaglandin E2 signaling. We aimed to block epileptogenesis using network-specific interventions as well as *via* a global anti-inflammatory approach using dexamethasone.

**Results:** (A) eFSE elicited a strong inflammatory response with rapid and sustained upregulation of pro-inflammatory cytokines. (B) Within minutes of the end of the eFSE, HMGB1 translocated from neuronal nuclei to dendrites, *en route* to the extracellular space and glial Toll-like receptors. Administration of an HMGB1 blocker to eFSE rat pups did not decrease expression of downstream inflammatory cascades and led to unacceptable side effects. (C) Prolonged seizure-like activity caused overall microRNA-124 (miR-124) levels to plunge in hippocampus and release of this microRNA from neurons *via* extra-cellular vesicles. (D) Within hours of eFSE, structural astrocyte and microglia activation was associated not only with cytokine production, but also with activation of the PGE_2_ cascade. However, administration of TG6-10-1, a blocker of the PGE_2_ receptor EP2 had little effect on spike-series provoked by eFSE. (E) In contrast to the failure of selective interventions, a 3-day treatment of eFSE–experiencing rat pups with the broad anti-inflammatory drug dexamethasone attenuated eFSE-provoked pro-epileptogenic EEG changes.

**Conclusions:** eFSE, a provoker of TLE-like epilepsy in rodents leads to multiple and rapid disruptions of interconnected glial-neuronal networks, with a likely important role in epileptogenesis. The intricate, cell-specific and homeostatic interplays among these networks constitute a serious challenge to effective selective interventions that aim to prevent epilepsy. In contrast, a broad suppression of glial-neuronal dysfunction holds promise for mitigating FSE-induced hyperexcitability and epileptogenesis in experimental models and in humans.

## Introduction

Febrile seizures (FS) are the most common seizure type in infants and young children and prolonged FS [febrile status epilepticus (FSE)] is associated with an increased risk of epilepsy in later life (1–6). The effects of FSE on neuronal structure and function are manifold and include persistent aberrant expression of critical neuronal genes, altered dendritic complexity and the development of abnormal excitatory synapses (7–11). However, epileptogenesis following FSE is complex. Genetic factors play a critical role in determining susceptibility to FSE and progression of epileptogenesis following FSE (3, 12–14). Mechanistically, glial cells are activated by prolonged FS and their release of inflammatory mediators may contribute to the pathogenesis of epilepsy (15, 16). Indeed, pre-clinical models of prolonged FS have identified rapid and persistent microglial and astrocyte activation following FSE and persistent upregulation of pro-inflammatory molecules including cytokines and prostaglandins (17–22).

The neuronal and glial response to human and experimental FSE and subsequent contribution to epileptogenesis are likely interdependent and involve a three-way neuronal-microglial-astroglial communication, activation and feedback (23, 24). Neuronal-glial cross-talk can take place in many ways and influence function accordingly (25–29). Recently, miRNAs were identified as strong candidate mediators of the communication among a number of glial cell types and neurons: miRNAs are abundant components of extra-cellular vesicles (ECVs), which upon generation in one cell type are released and can traverse the extracellular space and enter cells of other types (30–32). This type of intercellular communication takes place bidirectionally between neurons and glia (both microglia and astrocytes) and also between microglia and astrocytes (30–34). ECV miRNA content was recently profiled during epileptogenesis and found to differ significantly in mouse brain following prolonged seizures (35–37). Once miRNA containing ECVs dissolve and release their cargo, miRNAs may influence the cellular machinery of their new host cell (38–40). Specifically, because miRNA modulate protein translation by repressing target mRNAs, they can provoke large scale changes in the repertoire of genes translated. In the context of epilepsy and epileptogenesis, the cellular origin of ECVs released during epileptogenesis and how they influence the function of recipient cells remains unknown.

miRNA transported *via* ECV are just one of a host of communication networks among neurons and glia that are initiated by epilepsy-inciting events including FSE. FSE rapidly initiates the subcellular translocation of the damage-associated molecular pattern (DAMP) molecule HMGB1 and subsequent cellular release from neurons which may influence inflammatory mediators on surrounding cells including Toll-Like receptors (TLRs) and RAGE receptors (16, 41, 42). The translocation of HMGB1 from the nucleus to the dendritic compartment of cells occurs rapidly following eFSE (43). This release and interaction may activate microglia and initiate large-scale inflammatory responses (20, 41, 43–47). Structural and molecular changes in microglia occur rapidly and persist throughout epileptogenesis following eFSE but how FSE induces the activated microglia phenotype remains elusive. However, the rapid neuronal DAMP molecular cascade represents an enticing target for intervention because it may initiate microglial and astrocytic activation and inflammatory consequences.

A number of cytokines have been shown to be involved in both human FSE (15) and experimental FSE and its consequences. These include interferon (48), Il-1β (49, 50) and others. In other types of prolonged seizures, prostaglandin and specifically EP2 have been implicated (51–53). Yet, the effects of FSE and other epilepsy-inciting events on neuronal-glial communication remain understudied, and which lines of communication fail or are enhanced during epilepsy development is unclear. A greater understanding of neuron-glial communication is critical because the mediators represent potential therapeutic targets. In the present study we use a multi-pronged approach to interrogate several neuronal-glial interactions and determine how they are affected by eFSE. We then tested whether intervention in these processes ameliorates eFSE-related development of spontaneous seizure development (epileptogenesis).

## Methods

### Experimental Overview

**Figure 1:**
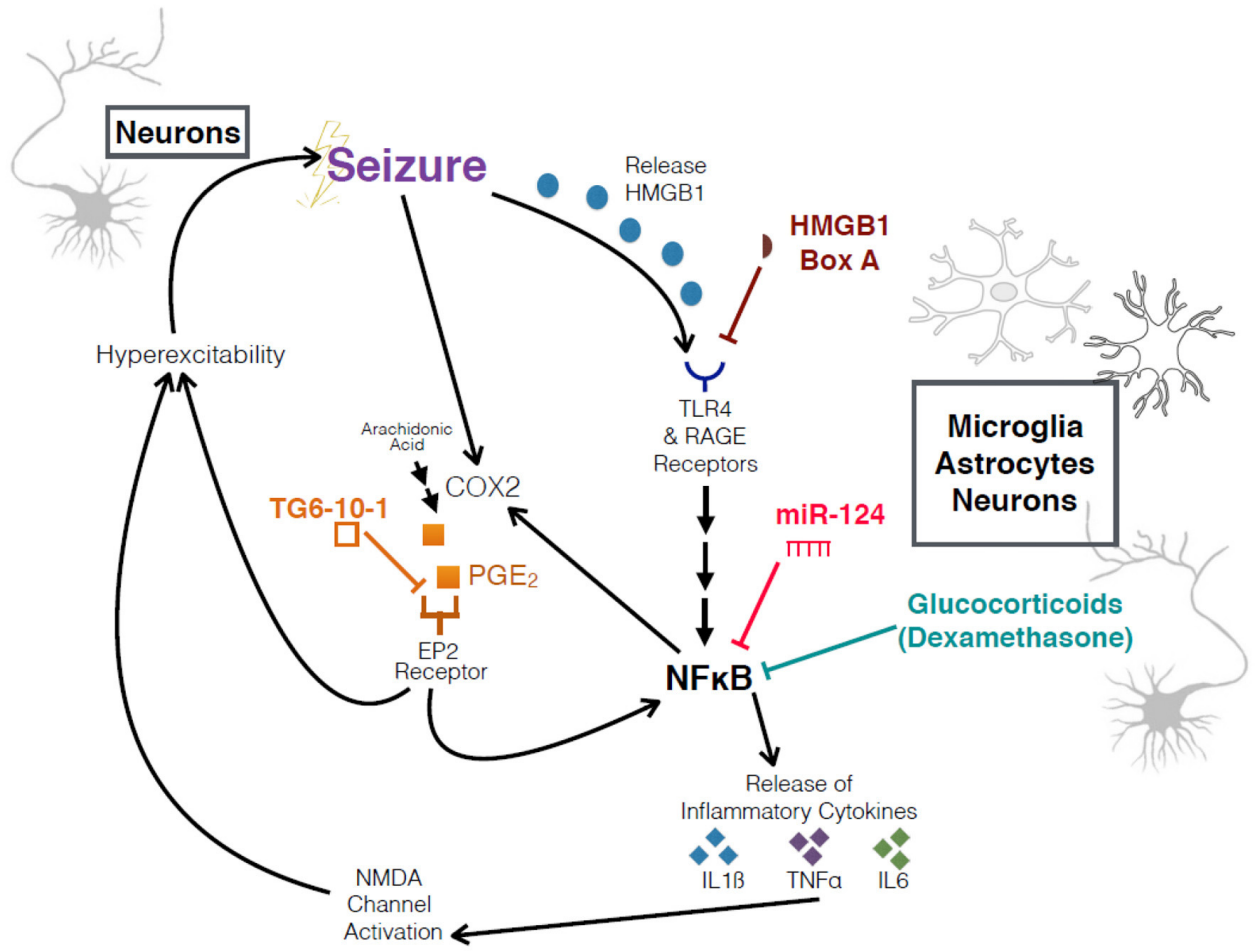
Schematic of interrogated pathways and experimental interventions. Overview of relationship between seizures, HMGB1, COX2, the inflammatory cascade, and an increase in hyperexcitability. HMGB1 Box A and TG6-10-1 are both competitive antagonists at their respective receptors.

The goal of the study was to identify the mechanisms by which eFSE triggers activation of glial cells in the brain and whether these signals originate in neurons. We also sought to explain the role of inflammation in the epileptogenesis that follows eFSE and whether targeting inflammation (either *via* targeted or global mechanisms) can block or ameliorate the subsequent epilepsy (Figure 1). To achieve these goals, we used a combination of *in vivo* models of FSE as well as *in vitro* hippocampal slice cultures in which we induce seizure-like events. Four experiments were conducted.

Experiment 1 characterized the inflammatory cascade elicited by eFSE at a number of important timepoints using qPCR quantification. We also measured and demonstrate the rapid subcellular translocation of the HMGB1 signaling protein which provokes microglial activation by binding to surface TLRs and RAGE receptors using immunohistochemical methods. Finally, in experiment 1 we examined whether blocking HMGB1 interaction with TLRs can reduce eFSE evoked inflammation.

Experiment 2 used organotypic hippocampal slice cultures to examine the mechanism by which the neuronal-specific miR-124, mediates microglial activation following prolonged neuronal activity. To determine whether miR-124 is released from neurons we measured the levels of miR-124 in culture media following seizure-like events using qPCR quantification. We also isolated ECVs and measured the levels of encapsulated miR-124 following seizure-like events to understand whether neuronal release is facilitated *via* ECVs.

Experiment 3 examined the effect of *post-hoc* EP2 receptor antagonism using TG6-10-1 (an EP2 receptor antagonist) on spike series development. eFSE rats [postnatal day (P)10-11] received three doses of either vehicle or TG6-10-1 at 4, 22, and 30 h post eFSE. EEG electrodes were implanted on P21 and recording began 4 days later following recovery from surgery. Recording was performed over 60 days.

Experiment 4 examined the effects of DEX on microglia and astrocyte activation and number and on the development of spike series (a measure of aberrant hippocampal excitability). Immediately following eFSE cessation, control and eFSE rats received injection of either vehicle or DEX. Hippocampal microglial activation and number were assessed using P2Y12 staining while astrocytes were identified using GFAP as a marker. As before, EEG was recorded from P21 over the course of 60 days to identify the emergence, frequency, and duration of spike series.

### Animals

Male Sprague Dawley rats were used for both *in vivo* and *in vitro* experiments. All experiments were performed with approval from the institutional animal care committee and conformed to National Institute of Health guidelines. Group sizes were determined *a priori* and animals were randomly assigned to experimental groups.

### Prolonged Febrile Seizure Induction

Experimental febrile status epilepticus (eFSE) was induced in male Sprague Dawley rats on post-natal day 10 or 11 (P10-11) as previously described (22, 50, 54). Briefly on P10-11 rats were weighed and pre-procedure weight was recorded. To prevent hyperthermia-related burns a thin wooden splint was attached to the tail and wrapped with a gauze. Ears and paws were then covered with a hydrating glycerin-based ointment. In pairs, rats were then placed in a 3 L flask and a warm stream of air was directed at them from a fixed height to elevate core body temperature gradually. The beginning of seizure was recorded as the onset of freezing behavior followed by chewing automatisms. Upon seizure onset a delicate rectal thermometer probe was used to measure core body temperature every 2 min to ensure body temperature remained between 39.5 and 41°C. Core body temperature was maintained at an elevated temperature for 60 min. Rats were then briefly immersed in room temperature water to restore normal body temperature and returned to home cages.

### Treatment With HMGB1 Box A

To block the pro-inflammatory action of HMBG1 on TL and RAGE receptors, we gave a cohort of animals intracerebroventricular (ICV) infusions of HMGB1 Box A, a direct antagonist. There were four groups studied: Naïve Control (Naïve-Ctrl; no eFSE, no infusion; *n* = 8), Control Vehicle (Ctrl-Vehicle; *n* = 5), eFSE-Vehicle (*n* = 7), and eFSE HMGB1 Box A (eFSE-Box A, *n* = 7). The vehicle and HMGB1 Box A groups, immediately following eFSE, were given ICV injections of 2.5 μl of either vehicle (sterile 0.9% saline) or HMGB1 Box A (3 μg/μl in sterile normal saline, for 7.5 μg/hemisphere), ICV dose of HMGB1 Box A was administered to each hemisphere, and then pups were sacrificed and hippocampal and amygdalar tissue samples collected either 3 or 6 h after infusion to measure HMGB1 signaling cascades. A third experimental cohort was generated to examine the longer-term inflammatory effects of HMGB1 Box A treatment at 24 h after eFSE. This experiment was aborted because the majority of rats that received HMGB1 Box A treatment were found to have rectal bleeding the following day, a side effect which did not occur following eFSE alone or with ICV-vehicle treatment.

### Treatment With TG6-10-1

TG6-10-1 was generously provided for this study by Professor Ray Dingledine and Emory University and used to block the Prostaglandin EPE2 Receptor. The TG6-10-1 was dissolved to a concentration of 0.25 mg/mL in the vehicle, a mixture of 10% dimethyl sulfoxide (DMSO) (molecular biology grade, Sigma-Aldrich, St. Louis, MO), 50% polyethylene glycol 400 (Sigma-Aldrich, St. Louis, MO), and 40% ultra-pure water (HyClone Water, ThermoFisher Scientific, Hampton, NH). All animals received 20 μl intraperitoneal injections of either the drug or the vehicle at 4, 22, and 30 h after the start of eFSE (Ctrl + Veh *n* = 5; eFSE + Veh *n* = 7; eFSE + drug *n* = 9).

### Dexamethasone Administration

Immediately following eFSE cessation, rats randomized into the dexamethasone group (Ctl-DEX, eFSE-DEX) were given an intra-peritoneal (i.p.) injection of dexamethasone (3 mg/kg; Sigma-Aldrich). Aldosterone was also administered with dexamethasone *via* sub-cutaneous (s.c) injection (0.2 μg/100 mg; Acros Organics) to provide mineralocorticoid functional support (55). This dose had been shown to fully suppress inflammation and employed a short-tapered treatment course to avoid many of the side effects (56–58). Rats received tapering doses of DEX (together with aldosterone) over 48 h as follows: 1.5 mg/kg DEX 24 h after eFSE; 0.75 mg/kg DEX 48 h after eFSE. Aldosterone was continued for the next 4 days (7 treatments total), to provide mineralocorticoid support during the potential suppression of the rats' adrenals. Vehicle controls consisted of i.p. saline. For the analyses reported here, group sizes were between 5 and 8.

### Electroencephalogram (EEG) Recording and Analysis

Following eFSE and respective treatments, bilateral hippocampal electrodes to bilateral dorsal hippocampus were surgically implanted (AP: −2.2 mm, LR: ±1.9 mm, Depth: 2.7 mm from bregma). Four days after recovery from surgery, Video-EEG recording began, using a tethered system with synchronized video recording (PowerLab data acquisition hardware, Bio Amplifiers, and LabChart 7 and 8 software, AD Instruments). Rats were recorded on a rotating basis for up to 60 days, with each recording period lasting at least 48 h. After recording, the EEG recording data and videos were analyzed for aberrant spike series by experienced investigators without knowledge of treatment groups.

The criteria used to identify spike series from the EEG data were: (1) Spikes must be clearly detectable from the background, with peak duration between 20 and 70 ms. (2) The spikes must have a biphasic shape with an amplitude of each peak at least two times higher than the baseline. (3) The slow waves occurring before the peaks need to be stable without any exhibiting significant movements (i.e., normal grooming) that would produce excessive artifacts. (4) At least four adjacent spikes with stable baseline are required to form a single spike series. (5) Excluded artifact based on video recording when animal was eating or scratching.

### Organotypic Hippocampal Slice Culture

Organotypic hippocampal slice cultures were prepared and maintained as previously described (59). Briefly we followed guidelines previously established using the interface method (60). Slices were prepared from rat pups on postnatal day 8 [Day *in vitro* 0 (DIV0)]. Rat pups were decapitated, brains removed, and hemispheres were separated. Three hundred micrometer thick slices were prepared on a McIlwain chopper and the hippocampus was dissected on ice cold prep media [MEM (Gibco), L-Glutamine (Gibco), Hepes Buffer (Fisher Scientific), Magnesium sulfate (Sigma), and cell culture grade water (GE Healthcare)] in a laminar flow hood. Slices were then maintained on 0.4 μm, 30 mm diameter cell culture inserts (Merck Millipore, Cork Ire) in six well plates with media containing MEM, HBSS (Gibco), L-Glutamine, Magnesium sulfate, Sodium bicarbonate (Gibco), Hepes, heat inactivated horse serum (Gibco), ascorbic acid (Sigma) and cell culture grade water. Slices were maintained in a CO_2_ enriched incubator (Thermo). On DIV 2 seizure-like activity was induced by transferring inserts to plates containing media supplemented with 6 μM KA (Abcam) and incubated for 3 h before being transferred to a new plate containing fresh media. Slices were harvested and media collected for analysis at 24 h post-seizure-like activity. Each well contained three slices, each from different pups. Slices from each well were pooled to make one sample. Each group contained three samples.

### Exosome Purification

Exosomes were isolated from 1 ml of media using the ExoQuick kit according to the manufacturer's instructions with modifications for volume differences. Briefly, the collected media was centrifuged to pellet cellular debris. The supernatant was removed to a fresh tube and Exoquick solution was added. The tubes were inverted and then incubated at 4°C overnight. Exosomes were then pelleted by centrifugation. The supernatant was aspirated and the exosome containing pellet was resuspended in lysis buffer. Hundred percentage of ethanol was added to the resuspended exosomes and the sample was transferred to an Exoquick RNA spin column. Exosome-derived RNA binds to the column and was washed and purified further with ethanol-based washes. The column was then transferred to a collection tube and RNA was captured by passing elution buffer through the column. The eluted RNA quantity and quality was measured using a nanodrop and stored at −80°C until required for downstream analysis.

### qPCR

Gene expression was measured as previously described (61). Using RNase free-dissection tools whole hippocampi were removed from rats and immediately stored at −80°C until use. Total RNA was isolated using the mirVana RNA isolation kit without small RNA enrichment according to the manufacturer's protocol. Quality and quantity of RNA was measured using a nanodrop with 260:280 and 260:230 values between 1.8 and 2.2 being considered acceptable for downstream analysis. cDNA libraries were then generated using a random hexamer reverse transcriptase approach. Analysis of the PCR product was performed using SYBR Green quantification on a Roche Lightcycler 96 well plate. Samples were analyzed in triplicate and normalized to a housekeeping gene using specific axon-spanning primers. For miRNA quantification and from exosomes specific stem loop primers were used for cDNA synthesis (ThermoScientific rno-miR-124-3p assay ID 001182). When comparing miRNA levels from exosomes miRNA levels were normalized to a spike in control. Quantification was performed using the ΔΔCt method.

### Immunohistochemistry and Cell Counting

For all procedures, rats were deeply anesthetized with pentobarbital and transcardially perfused with 4% paraformaldehyde (PFA) at desired time points post eFSE. Brains were removed and post-fixed in 4% PFA for 90 min. Brains were then cryoprotected in 30% sucrose, rapidly frozen and stored at −80°C. Thirty-micron sections of dorsal hippocampus were obtained on a cryostat and stored in anti- freeze at 4°C until use. Serial sections were blocked in 10% normal goat serum and 0.03% Triton-x in 1x PBS for 1 h at 4°C. Primary antibodies were incubated in 4% Normal Goat Serum with 0.03% Triton-x overnight at 4°C. The following antibodies were used: rabbit anti-HMGB11:1,000 (Abcam), mouse anti-GFAP 1:3,000 (Millipore), mouse anti-IBA1 or P2Y12 1:4,000 (Wako). Sections were washed with 1x PBS and the reaction product was visualized using a 3,3′-diaminobezidine. Colocalization of cell markers with HMGB1 was achieved by co-incubating rabbit anti-HMGB1 1:1,000 (Abcam) with the following antibodies: mouse anti- NeuN (Chemicon), mouse anti-GFAP 1:3,000 (Millipore) and mouse anti-CD11b (ABD Serotec). After 24-h incubation, sections were washed in 1x PBS and then incubated in the appropriate secondary antibodies conjugated to Alexa Flour 568 or Alexa Flour 488. Colocalization was visualized using confocal microscopy.

For quantification of activated microglia and astrocytes, group sizes were 5–8. Assessment of cell numbers in hippocampal CA1, CA3 and hilus was achieved by boxing off 1,000 by 500 μM areas of each studied region. First, all P2Y12 or IBA1+ (or GFAP) positive cells in the defined region were counted and compared among groups. Activated microglia were identified as IBA1+ cells that had large, dark soma and few short processes that were thicker in appearance than non-activated microglia. Microglia activation is presented as the percent change in activated microglia over the total number of P2Y12 or IBA+ cells. Quantification of GFAP+ cells was achieved by using the same hippocampal regions and area delineations. GFAP+ cells were counted and compared among groups.

Group sizes for the HMGB1 translocation studies were three per group per timepoint. Two representative and matching section per animals were employed. HMGB1 quantification was accomplished by counting all HMGB1+ cells in a 100 by 500 μM region of CA1. Translocation of HMGB1 protein was identified by the presence of immunoreactivity outside the nucleus, i.e., in the somatic cytoplasm and in the processes of cells. Change in HMGB1 translocation is presented as the percent change in translocated cells over the total number of HMGB1+ cells.

### Statistical Analyses

All analyses were performed without knowledge of treatment assignment. Comparisons of two groups were analyzed using standard *t*-test while analyses comprising more than two groups were performed using one- or two-way ANOVA. Outliers for all qPCR data were analyzed based on Rout Test Q = 5% and removed prior to the completion of any analysis. Statistical significance was set at less than or equal to 0.05.

## Results

### Increased Expression of Inflammatory Cytokines, Including Prostaglandins, Following eFSE

**Figure 2:**
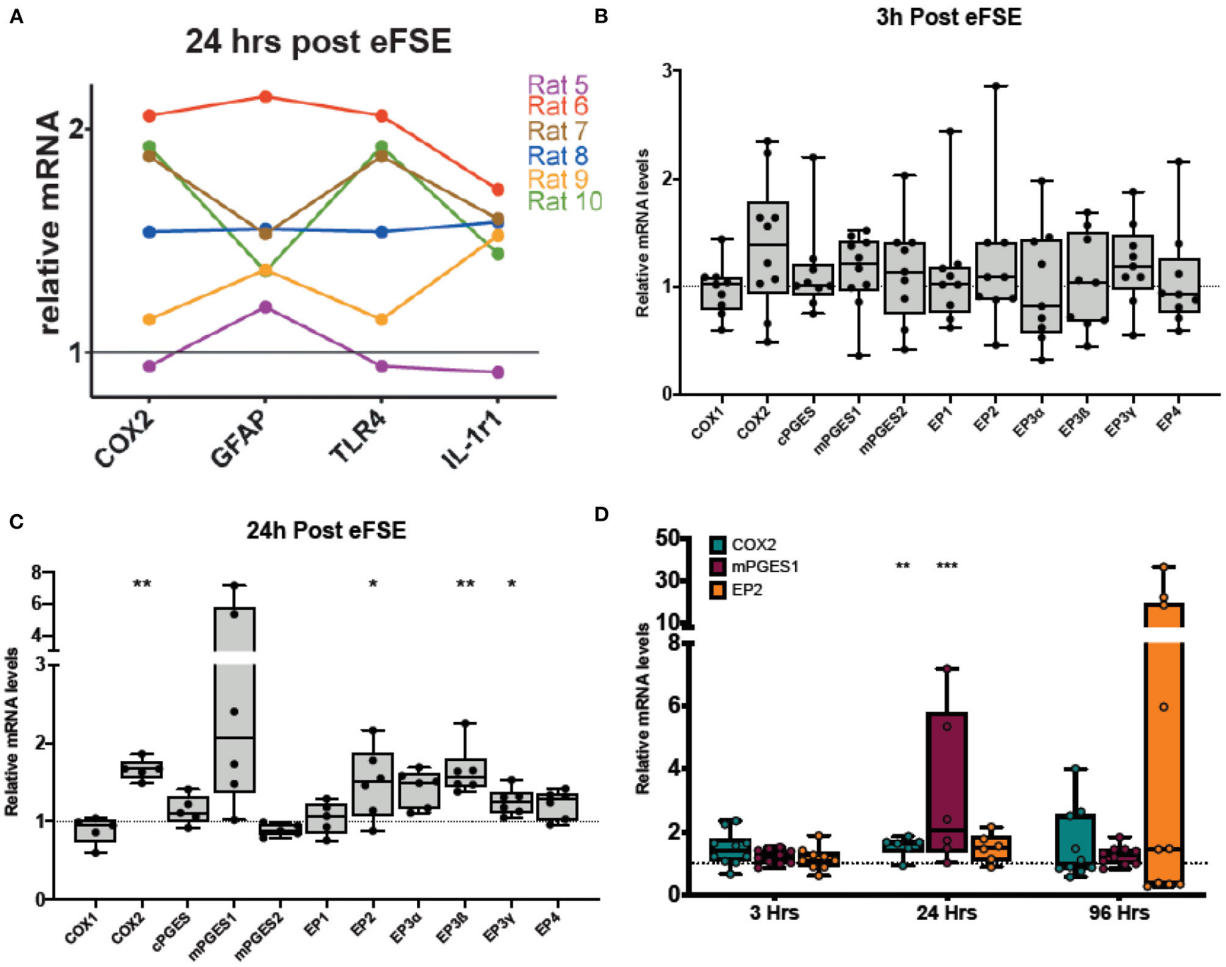
Rapid and persistent activation of inflammatory cascades following eFSE. **(A)** Twenty-four hours after eFSE, COX2, GFAP, TNFα, and IL-1r1 levels were significantly increased at the mRNA level. When the expression profile of an individual rat is examined across these mediators, trends in expression appear. **(B)** Examination of inflammatory mediators including those involved in the prostaglandin-mediated inflammatory pathway at early timepoints suggests the inflammatory cascade is rapidly induced by eFSE as early as 3 h post-insult in a subset of rats but not all. **(C)** Examination of the same set of inflammatory mediators at 24 h showed persistent dysregulation and significant disruption of COX2 and downstream signaling components. **(D)** qPCR analysis of COX2, mPGES1 and EP2 at 96 h post eFSE demonstrates persistent disruption of both COX2 and EP2 in a subset of rats (~40%) consistent with our previous findings. **(A)** Adapted from (20). *p < 0.05; **p < 0.01; ***p < 0.001.

Our previous work has revealed that a subset of rat pups that undergo eFSE have a coordinated increase in a wide variety of inflammatory markers (Figure 2A). These same rats also had amygdalar MRI signal changes which predicted later epilepsy (20). In addition to traditional cytokines, we measured the enzyme COX2 because it was the most strongly correlated with the T_2_ signal changes and had the most long-lasting increase in expression, encouraging further work investigating the COX2-prostaglandin pathway.

To investigate the COX2-prostaglandin inflammatory cascade, we measured the expression of mRNA levels of COX1, COX2, the three prostaglandin E synthase enzymes (cPGES, mPGES1, and mPGES2), as well as the prostaglandin E_2_ (PGE_2_) receptors (EP1, EP2, EP3α, EP3ß, EP3γ, EP4). At 3 h following eFSE, there was no significant increase in expression of the cascade (Figure 2B, unpaired *t*-test, *p* > 0.05 for all). At 24 h after eFSE, we found significantly increased expression of COX2, EP2, EP3ß, EP3γ, with a subset of the rats having markedly high expression of mPGES1 (Figure 2C, unpaired *t*-test, COX2: *p* = 0.002, t = 4.21, df = 9; mPGES1: *p* = 0.07, t = 2.01, df = 10; EP2: *p* = 0.04, t = 2.33, df = 10; EP3ß: *p* = 0.003, t = 4.48, df = 10, EP3γ: *p* = 0.01, t = 3.024, df = 10). These are all elements of the PGE_2_ pathway, which has been investigated for its role in epileptogenesis, specifically the EP2 receptor. Thus, we next analyzed the expression of COX2, mPGES1, and EP2 at 96 h after eFSE. While not statistically significant when grouped together, a subset of rats had very high expression of EP2 at 96 h, much higher levels than any of the inflammatory cytokines previously analyzed (Figure 2D, EP2: *p* = 0.21, t = 1.31, df = 13), This selective increase in a subset of animals across inflammatory markers (Figures 2B–D) is consistent with prior findings in the animal studies of eFSE (20) and with human data that only a subset of children are at risk of developing epilepsy following prolonged febrile seizures (1, 3, 5). Thus, these findings encouraged investigation of inflammation and EP2 blockade to prevent the development of abnormal hyperexcitability.

### HMGB1, a Potent Antecedent of Cytokine Expression Rapidly Translocates During eFSE, but Its Blockade Does Not Decrease Inflammation

**Figure 3:**
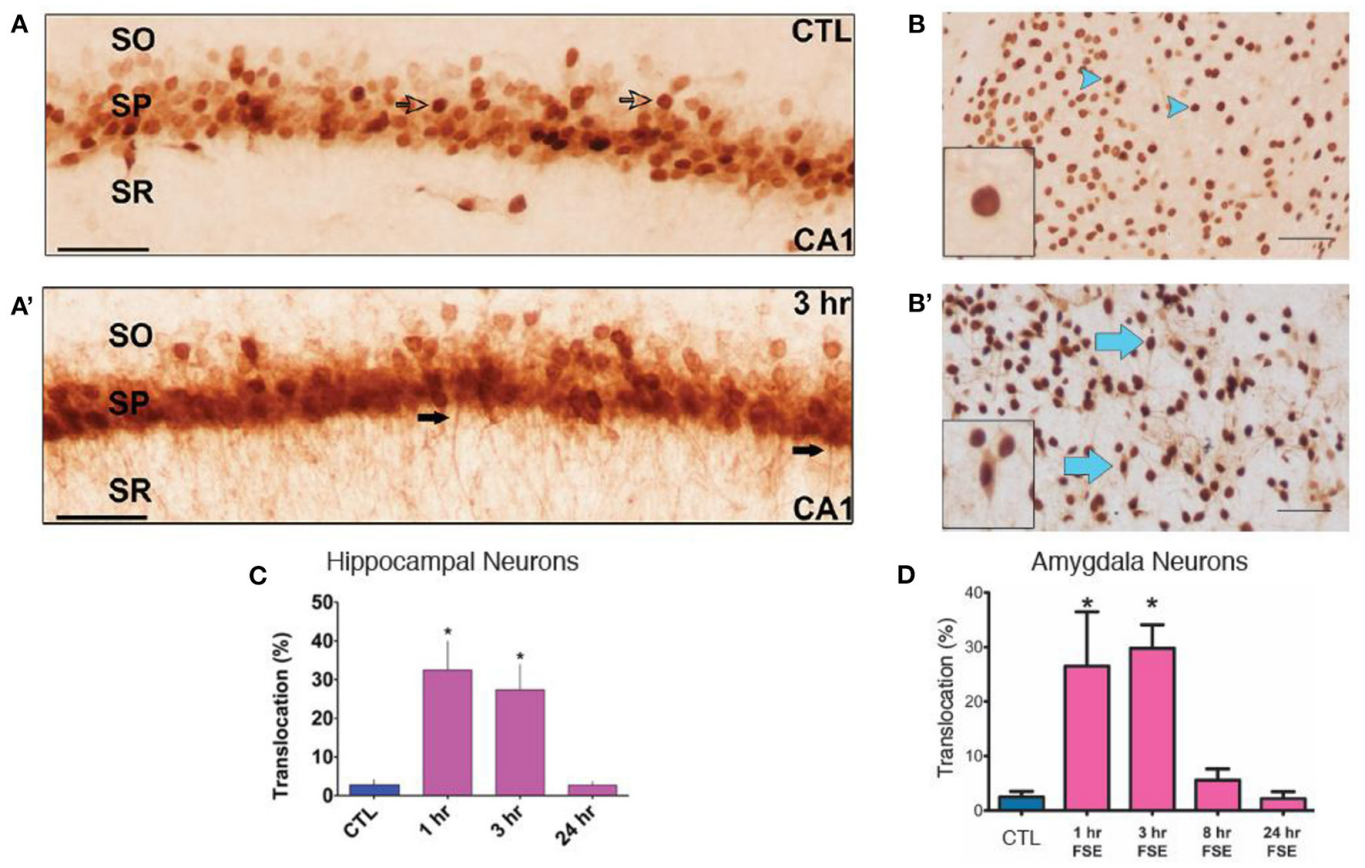
HMGB1 Translocation in the Amygdala and Hippocampus following experimental Febrile Status Epilepticus (eFSE). **(A)** In control (CTL) animals, HMGB1 is confined to the nucleus of cells (open arrows) in area CA1 of dorsal hippocampus. **(A′)** Three hours after the end of eFSE, HMGB1-IR is seen in the processes of neurons (closed arrows), indicating translocation of HMGB1 from the nucleus. **(B,B′)** Differences in HMGB1 localization between normothermic control (NT-C) and experimental FSE rats. Arrowheads indicate nuclear HMGB1 and arrows indicate cytoplasmic (translocated) HMGB1. **(C)** Quantification of HMGB1 immunocytochemistry as the percentage of HMGB1-IR cells with translocation over total HMGB1-IR cells shows a significant increase in the percentage of HMGB1 translocation 1 and 3 h after eFSE with a return to control conditions by 24 h. Data are presented as the mean ± SEM. Scale bars, 100 μm. *Statistically significant at *p* < 0.05. CTL, Control; SO, stratum oriens; SP, stratum pyramidale; SR, stratum radiatum. **(A,A′,C)** Adapted from (20). **(B,B′,D)** Adapted from (43), and *n* = 3 per group per timepoint.

In view of the robust increase of cytokine expression rapidly after eFSE, we examined the putative upstream mechanisms. Translocation of the neuronal danger signaling molecule HGMB1 from the nucleus to the cytoplasm and its release into the extracellular space is known to initiate cytokine expression by activating TLRs on glial cells (25, 62–64). Indeed, translocation of HMGB1 from the nucleus into the cytoplasm of neurons in both the amygdala and hippocampus of rats that underwent eFSE was found in prior work (Figures 3A,A′,B,B′). There was a significant increase in translocation 2 and 4 h after the start of eFSE within the amygdala at 1 and 3 h after eFSE but had returned to normal by 8 h (Figure 3D) (43). This same temporal pattern was seen in the hippocampi of rats that experienced eFSE (Figure 3C) (20).

**Figure 4:**
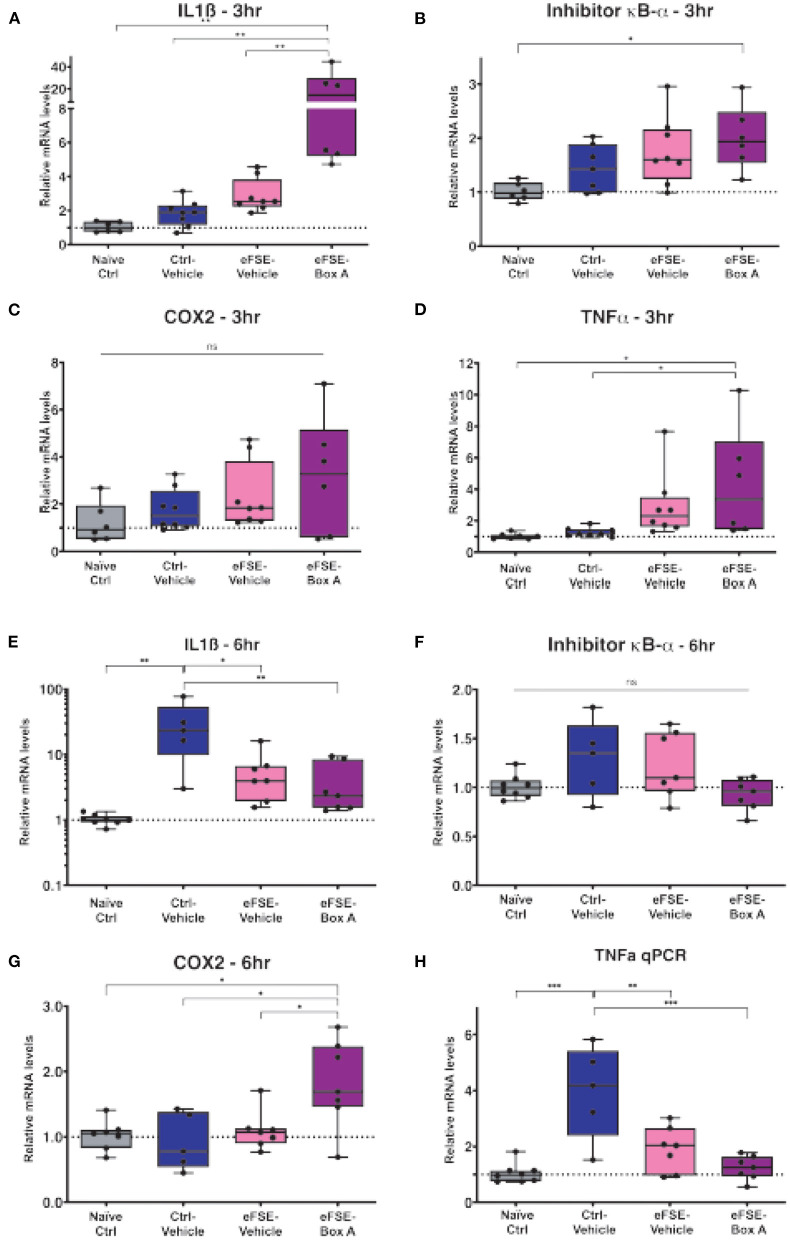
HMGB1-inhibition does not reduce eFSE-evoked inflammation. **(A–D)** qPCR analysis of pro-inflammatory cytokines at 3 h post eFSE with or without HMGB1 inhibition. HMGB1 inhibition actually increased levels of pro-inflammatory cytokines. **(E–H)** qPCR analysis of pro-inflammatory cytokines 6 h post eFSE and treatment with veh or Box A. While infusion of Veh alone even in control rats was sufficient to induce inflammation Box A delivery was not effective at blocking inflammatory signaling post eFSE. *p < 0.05; **p < 0.01; ***p < 0.001.

Based on this, we investigated if HMGB1 blockade in neurons would decrease eFSE-provoked microglial activation and cytokine expression. Following eFSE, we administered ICV injections of HMGB1 Box A, a direct HMGB1 antagonist. We found that at 3 h after eFSE, HMGB1 Box A treatment did not significantly reduce the expression of pro-inflammatory cytokines including IL1ß, COX2, TNFα, and Inhibitor κB-α (IκB-α; a measurement of NFκB activity, a direct downstream target of HMGB1) (Figures 4A–H, one-way ANOVA with Bonferroni correction for multiple comparisons, IL1ß: naïve-ctrl vs. eFSE-Box A *p* = 0.003, ctrl-veh vs. eFSE-Box A *p* = 0.003, eFSE-veh vs. eFSE-Box A *p* = 0.005; IκB-α: naïve-ctrl vs. eFSE-Box A *p* = 0.014; COX2: all adjusted *p* > 0.05; TNFα: naïve-ctrl vs. eFSE-Box A *p* = 0.04). Indeed, rather than suppressing cytokine expression, HMGB1 Box A actually increased expression of several inflammatory molecules (Figures 4A,B,G). Notably, administration of HMGB1 Box A to immature rats led to rectal bleeding in some rats at the 24 h timepoint, leading to a termination of this line of work. The lack of success in blocking pro-inflammatory cytokines combined with unacceptable side effects led us to conclude that interfering with HMGB1 *via* the direct antagonist HMGB1 Box A was not an encouraging candidate to prevent the long-term consequences of eFSE.

### MiR-124 Is Released From Neurons via ECVs Following Seizure-Like Activity

**Figure 5:**
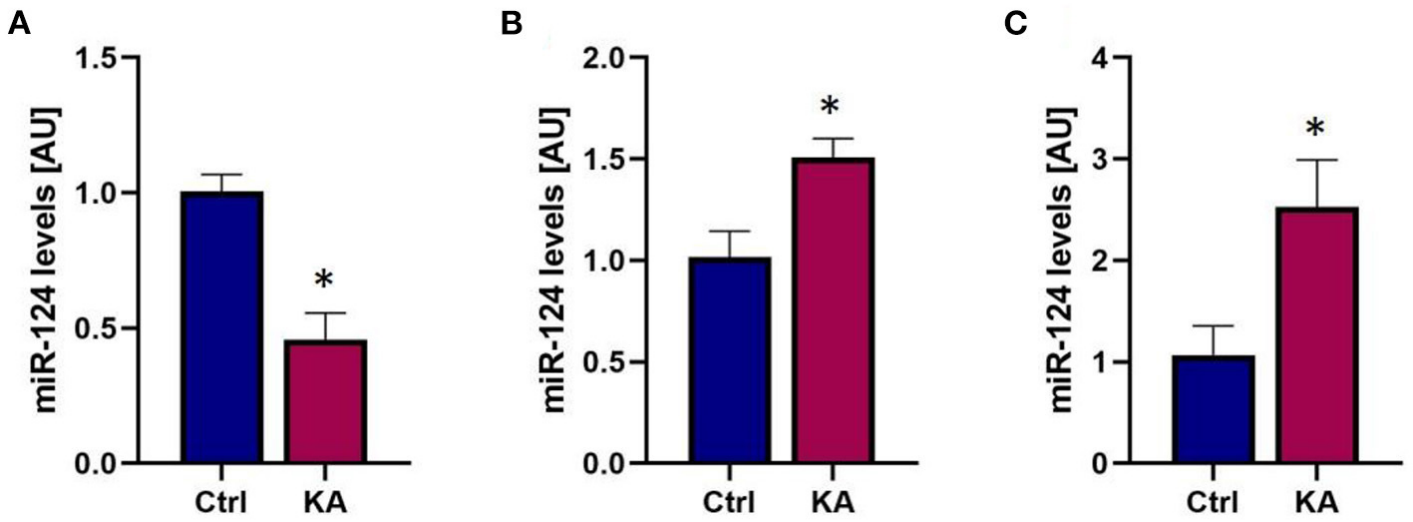
Seizure-like activity reduces total miR-124 levels but promotes miR-124 packaging into ECVs and release. **(A)** qPCR analysis reveals rapid reduction of miR-124 levels in organotypic hippocampal slices following seizure-like events. **(B)** miR-124 levels in culture media increase following seizure-like events. **(C)** Analysis of miR-124 levels from exosomes isolated from culture media shows enrichment of miR-124 in exosomes following seizure-like events. *p < 0.05.

Inter-cellular communication can take place when ECVs containing miRNAs originating from one cell type are released and taken up by a recipient cell (30, 38, 65, 66). The released ECV cargo can then influence its new environment. miRNA-124 is produced almost exclusively in neurons in response to intense activity such as seizures (61, 67), yet has been reported to influence microglial activity and cytokine release (66). To test whether miRNA-mediated neuronal-glia communication may occur following seizures we induced seizure-like activity in organotypic hippocampal slice cultures to approximate the type of seizure activity induced by eFSE. We used kainic acid KA at a dose which does not induce cell death (59), and that results in prolonged seizure-like network activity for the duration of exposure (68). We then measured the levels of miR-124 in slices and in the surrounding medium to measure exported miRNA levels (Figures 5A,B). We found a rapid reduction in miR-124 levels in the hippocampal tissue, recapitulating our prior findings both *in vivo* and *in vitro* (61). Notably, miR-124 was readily detected in the medium. To determine whether the mechanism of release was *via* ECV expulsion, we then isolated ECVs from the medium and quantified miRNA content. We found that miR-124 was enriched within ECVs, and particularly from slice cultures treated with KA (Figure 5C).

These findings supported the impetus to consider miR-124 as a potential intercellular communicator between neurons and glia that might play a role in the epileptogenesis that follows eFSE (61).

### Blockade of Prostaglandin E_2_ (PGE_2_) at the EP2 Receptor Does Not Prevent Aberrant Hyperexcitability

**Figure 6:**
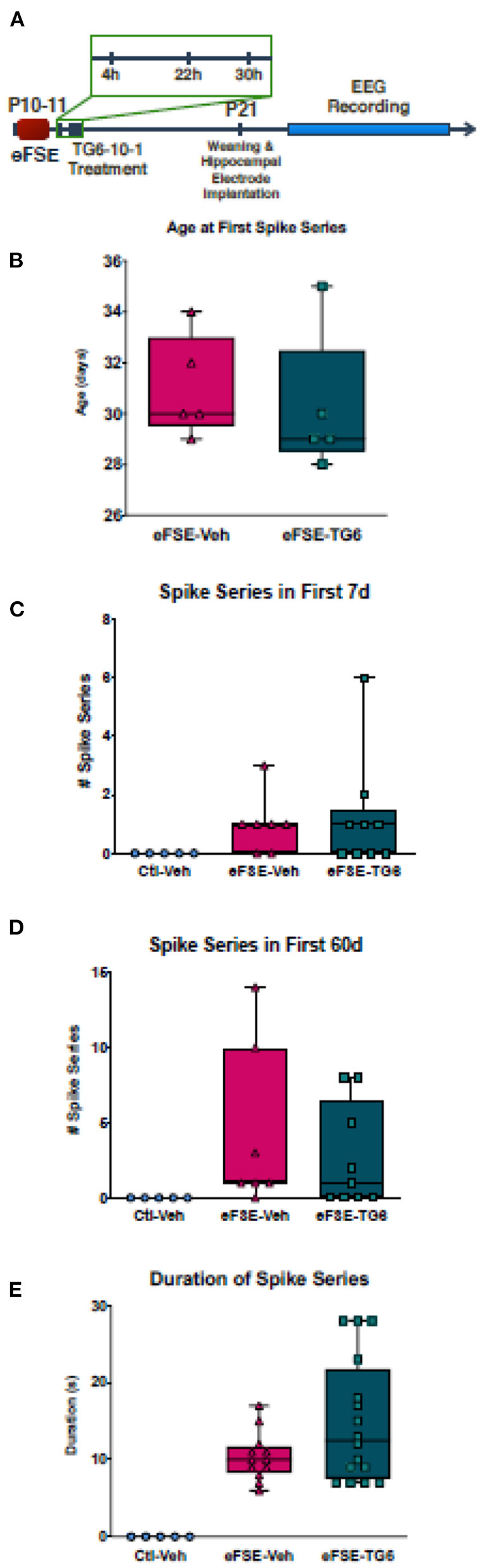
TG6-10-1 treatment does not ameliorate or alter eFSE-evoked epileptogenesis. **(A)** Schematic of experimental design. TG6-10-1 treatment failed to alter the latency to onset of spike series post eFSE **(B)**, the total number of aberrant EEG spikes recorded in the first 7 days **(C)**, the first 60 days **(D)** nor the duration of each spike series **(E)**.

As mentioned above, the neuroinflammatory molecules whose expression was induced by eFSE included members of the prostaglandin family, and especially the EP2 receptor. Therefore, we probed the potential contribution of PGE2, and specifically its signaling *via* the EP2 receptors (51, 53, 69). The small molecule inhibitor, TG6-10-1, is a direct antagonist of PGE_2_ at the EP2 receptor developed by Dingledine et al. (51). We administered TG6-10-1 to rats that had undergone eFSE at 4, 22, and 30 h post-eFSE and then recorded video-EEG from hippocampal electrodes to analyze spike series, a sign of aberrant hyperexcitability and precursor to epilepsy (22) (experimental design, Figure 6A). Consistent with previous work, none of the control rats that were recorded had spike series. There was a subset of rats within both the rats that underwent eFSE and received vehicle injections (eFSE-Veh) and those that received TG6-10-1 injection (eFSE-TG6) that had measured spike series. When analyzed, there was no effect of TG6-10-1 treatment on the age at onset of spike series (Figure 6B, unpaired *t*-test, *p* = 0.61), the number of spike series within the first week of recording (Figure 6C, one-way ANOVA with Bonferroni's correction for multiple comparison, eFSE-Veh vs. eFSE-TG6, *p* > 0.99), total spike series in 60 days of recording (Figure 6D, one-way ANOVA with Bonferroni's correction for multiple comparison, eFSE-Veh vs. eFSE-TG6, *p* > 0.99), or duration of spike series (Figure 6E, one-way ANOVA with Bonferroni's correction for multiple comparison, eFSE-Veh vs. eFSE-TG6, *p* = 0.18). This led us to definitively conclude that short term treatment with TG6-10-1 does not decrease the development of abnormal hyperexcitability following eFSE, and thus, is unlikely to prevent the development of eFSE-induced epilepsy.

### Global Suppression of Neuroinflammation Abrogates Pro-epileptogenic Neuronal Changes Following eFSE

**Figure 7:**
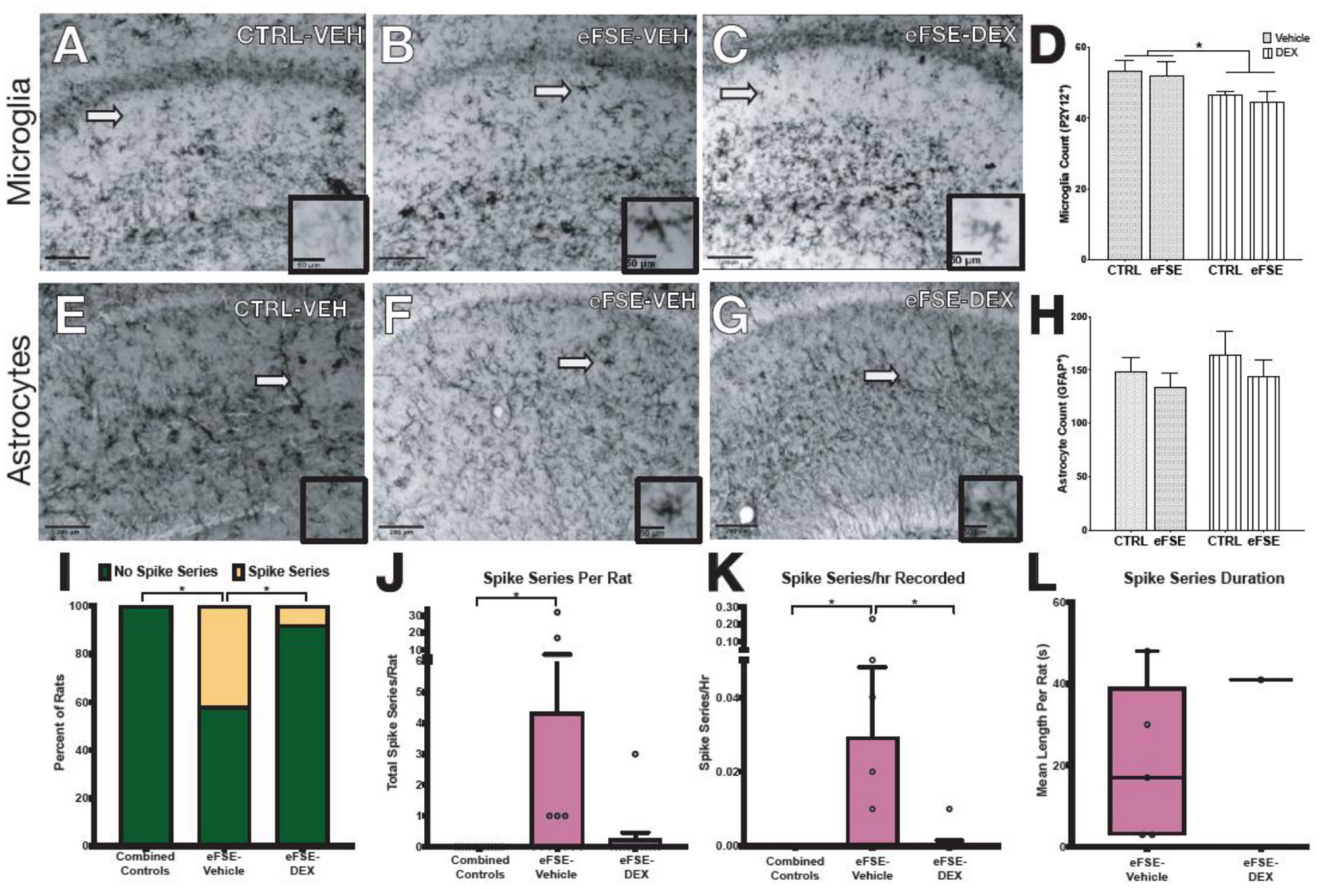
Dexamethasone reduces inflammation following SE and epileptogenesis-associated spike-series. **(A–D)** Representative photomicrographs of hippocampal microglia staining with P2Y12 in control-Veh **(A)**, eFSE-Veh **(B)**, and eFSE-DEX **(C)** plus quantification **(D)**. Hippocampal microglial counts revealed a significant reduction in the number of activated microglia in eFSE rats treated with DEX as compared to control rats treated with Veh or Dex or eFSE rats treated with Veh. No effects were detected when astrocytes were analyzed **(E–H)**. **(I)** DEX treatment significantly reduced the number of rats who developed electrographic spike series after eFSE. Overall DEX treatment reduced the total number of spike series identified in rats following eFSE **(J)**, the frequency of spike series recorded per hour **(K)**, and the duration of spike series which were detected **(L)**. Group sizes: *n* = 5–8, the figure is adapted from (22). *p < 0.05.

The experiments described above suggested that targeting individual neuron-glia communication cascades to eliminate eFSE-induced epileptogenesis would not be successful. Yet, the evidence for contribution of neuroinflammatory mediators to epileptogenesis is strong. To address this conundrum, we investigated whether a global anti-inflammatory drug would decrease the aberrant hippocampal network hyperexcitability that follows eFSE. We first examined whether dexamethasone (DEX), a synthetic glucocorticoid, given for 3 days following eFSE attenuated glial proliferation. DEX treatment following eFSE decreased the density of P2Y12+ microglia [Figures 7A–D, adapted from (22), two-way ANOVA; main effect of DEX, *p* = 0.016]. The drug had no effect on astrocyte number which was unaffected by the eFSE [Figures 7E–H, adapted from (22), two-way ANOVA; main effect of eFSE, *p* = 0.30; DEX *p* = 0.43]. We then utilized EEG to determine if DEX attenuated the eFSE-induced aberrant hyperexcitability, measured as spike series. Spike series classically precede the development of spontaneous seizures in eFSE rats that go on to develop epilepsy [(50, 70–72); see (22)]. Therefore, spike series can be used as a measure of abnormal hippocampal excitability in this context. DEX treatment reduced the proportion of rats with recorded spike series in as compared to their vehicle-eFSE littermates (Figure 7I, unconditional Barnard's exact test, control vs. eFSE-VEH, *p* = 0.0062; eFSE-VEH vs. eFSE-DEX, *p* = 0.031; control vs. eFSE-DEX, *p* = 0.37). DEX also reduced the mean number of spike series per rat (Figure 7J, Kruskal–Wallis ANOVA: mean rank difference, −8.083; *p* < 0.01; Dunn's multiple-comparisons test, eFSE-DEX vs. controls: mean rank difference, −1.53; *p* = 0.999; eFSE-VEH vs. eFSE-DEX: mean rank difference, 6.55; *p* = 0.067). DEX attenuated the mean spike series frequency (spike series/hour recorded) bringing the level down to that seen in controls (Figures 7K,L, CTL mean, 0 ± 0; eFSE-VEH mean, 0.029 ± 0.06; K-W ANOVA: K-W statistic, 9.71; mean rank difference, −8.29; *p* < 0.01; Dunn's multiple-comparison test, eFSE-DEX vs. eFSE-VEH: mean rank difference, 6.95; *p* = 0.046; eFSE-DEX vs. controls: mean rank difference, −1.35; *p* = 0.99) [Figures 7I–K, adapted from (22)].

## Discussion

The current study identifies a critical role for neuro-glial communication in epilepsy development caused by eFSE. This includes the rapid translocation of HMGB1 from neurons, which then initiates microglial activation by binding to glial surface TL and RAGE receptors. We also identify for the first time a direct release of neuronal miRNA-124 from neurons in response to aberrant network activity, which provides a mechanism for the previously suspected role for neuronal miRNAs in seizure-induced microglial activation (61). We attempted to ameliorate epileptogenesis following eFSE by targeting individual components of the inflammatory cascade, specifically HMGB1 and the EP2 receptor, but found that this approach was unable to block the development of inflammation and hyperexcitability, respectively. We therefore took a more global approach and blocked inflammation using DEX and found that this blunt force approach could in fact prevent the development of hippocampal hyperexcitability following eFSE. Together our findings highlight the complexity of inter-cellular communication following eFSE and show that a hammer rather than scalpel approach may be required to overcome the complexities of eFSE-induced epileptogenesis and to prevent the emergence of epilepsy.

We have previously seen a coordinated inflammatory response following eFSE, with rapid and persistent increase in pro-inflammatory cytokine levels including elevated IL1β, TNFα, COX-2, and Il-6 (20). Here we examined and found marked activation of pro-inflammatory cytokines but also the prostaglandin inflammatory cascade after eFSE. We observed persistently elevated EP2 receptor levels in a subset of eFSE rats. The EP2 receptor is upregulated in several animal models of epilepsy and prolonged seizures including the pilocarpine model of epilepsy and following pentylenetetrazole (PTZ) and diisopropofluorophosphate (DFP)-induced seizures (73, 74). FSE results in epilepsy in about 40% of cases (6, 75), and we see the same penetrance in rodent models of eFSE (43). Here, we see persistent activation of the prostaglandin in only a subset of rats suggesting that inflammatory processes are intrinsically linked to epilepsy development.

Our recent studies have found that eFSE rapidly promotes HMGB1 translocation from neuronal nuclei to the cytoplasm and the surrounding extracellular space where it binds surface TL and RAGE receptors on neighboring microglia, initiating an inflammatory response (43). Inhibiting HMGB1 signaling with Box A has previously been shown to be anti-convulsant when administered before a seizure challenge (25) to adult rodents; others reported that intra-nasal administration of recombinant HMGB1 reduced seizure threshold and promoted hyperthermia-induced seizures (46). In view of the above work, we sought to block HMGB1 signaling to reduce eFSE-evoked microglial activation and inflammation. We found that, within 3 h, HMGB1 antagonism with Box A actually increased the levels of specific pro-inflammatory cytokines including Il-1ß and COX2 which are known to contribute to eFSE-evoked epileptogenesis. These unexpected findings suggest that HMGB1 blockade may activate alternative or compensatory inflammatory pathways which in this case resulted in an even greater inflammatory response. This seems further plausible given the unacceptable side effects we detected at later time points in young rats treated with this drug.

While it is accepted that neuronal-glial communication plays a key role in the epileptogenesis evoked by many epilepsy-inciting events including FSE, the mechanisms by which neurons and glia communicate during epilepsy development are relatively unknown. Extracellular vesicles including exosomes have emerged as critical mediators of cell-cell communication and are enriched in the CNS. Detailed tracing studies now exist which follow ECV export from originating cells and subsequent uptake by surrounding cells (30, 76). Recent studies have profiled exosome miRNA content during epileptogenesis and found gross changes in exosomal miRNA content at specific disease stages (35–37). Our previous studies found that miR-124, despite residing in neurons could influence the inflammatory response when introduced to microglia (61). Here we find that seizure-like events promote miR-124 inclusion in ECVs which are released by neurons and may enter neighboring cells. Due to the neuronal specific nature of miR-124 we can conclude that ECVs containing miR-124 originate in neurons. We specifically used a non-excitotoxic dose of KA to evoke seizure-like events which suggests that ECV and miR-124 release from neurons is not due to seizure-induced neuronal apoptosis or necrosis. Instead miR-124 containing ECVs are actively exported from viable yet stressed neurons and may influence surrounding cells. Thus, we implicate miR-124 as a critical regulator of neuronal-glial crosstalk during epileptogenesis.

Having identified EP2 as heavily involved in the inflammatory milieu following eFSE we next sought to block EP2 using a highly specific inhibitor as a potential anti-epileptogenic approach (51). Surprisingly we found that inhibition of the EP2 receptor had no effect on the emergence of abnormal hyperexcitability induced by eFSE and likely would be ineffective at blocking epileptogenesis. Previous studies utilizing EP2 inhibition have successfully ameliorated the long-term effects of status epilepticus (52). These studies however have been mostly performed in adult models of epilepsy and this is further evidence that targeting individual inflammatory pathways in juvenile epilepsy may activate alternative inflammatory pathways in response or be insufficient to have meaningful effects on epilepsy development.

To test this, we next investigated whether using a less specific anti-inflammatory agent may ameliorate or alter eFSE-evoked epileptogenesis. We used the glucocorticoid dexamethasone (DEX) which effectively and broadly dampens inflammation and is used to treat many inflammation-related diseases. We found that administration of DEX following eFSE blocked microglial activation and proliferation in the hippocampus and also led to spike series development in much fewer animals, with most DEX treated rats not developing spike series at all. This suggests that anti-inflammatory approaches, at least for eFSE-induced epilepsy might be better treated with broad-acting anti-inflammatory drugs like DEX rather than those which target individual receptors or cytokines. It is also conceivable that etiology plays a large role and anti-epileptogenic strategies must be designed with this in mind.

Together our findings suggest that eFSE in rodents leads to multiple and rapid disruption of interconnected glial-neuronal networks with a likely role in epileptogenesis. The intricate, cell-specific and homeostatic interplays among these pathways constitute a serious challenge to effective selective interventions that aim to prevent epilepsy. In contrast, a broad suppressive measure of glial-neuronal dysfunction holds promise for mitigating FSE-induced hyperexcitability and epileptogenesis in experimental models and in humans.

## Data Availability Statement

The raw data supporting the conclusions of this article will be made available by the authors, without undue reservation.

## Ethics Statement

The animal study was reviewed and approved by University of California-Irvine Animal Care Committee.

## Author Contributions

GB, MG-C, KP, and RL performed experiments. TB, GB, and MG-C designed experiments. GB, MG-C, and TB wrote the paper. All authors contributed to the article and approved the submitted version.

## Conflict of Interest

The authors declare that the research was conducted in the absence of any commercial or financial relationships that could be construed as a potential conflict of interest.
